# Positive resection margins may not reflect the true margin in patients undergoing radical prostatectomy

**DOI:** 10.3892/ol.2014.2491

**Published:** 2014-09-01

**Authors:** NOBUKI FURUBAYASHI, TAKAHITO NEGISHI, YU HIRATA, KENICHI TAGUCHI, MOTOTSUGU SHIMOKAWA, MOTONOBU NAKAMURA

**Affiliations:** 1 Department of Urology, National Kyushu Cancer Center, Fukuoka 811-1395, Japan; 2 Department of Pathology, National Kyushu Cancer Center, Fukuoka 811-1395, Japan; 3 Institute for Clinical Research, National Kyushu Cancer Center, Fukuoka 811-1395, Japan

**Keywords:** prostate cancer, surgical margin, radical prostatectomy, ultrasensitive prostate-specific antigen, prostate-specific antigen nadir

## Abstract

The aim of the present study was to evaluate the hypothesis that a positive resection margin (RM1) of an excised specimen may not reflect the true margin in patients that have undergone radical prostatectomy (RP). Between September 2003 and March 2011, 370 Japanese patients underwent an antegrade RP at the National Kyushu Cancer Center (Fukuoka, Japan), however, 95 of these patients were excluded from the study due to a history of receiving hormonal therapy or insufficient preoperative clinical data. The incidence of biochemical failure (BCF) was evaluated using multivariate analysis, which revealed that the preoperative prostate-specific antigen (PSA) level, pathological tumor stage, RP Gleason score and a PSA nadir <0.008 ng/ml were significant predictors (P=0.0065, 0.0006, 0.0002 and <0.0001, respectively). By contrast, an RM1 was not found to be a significant predictor of BCF, while the parameter with the highest hazard ratio (HR) was a PSA nadir <0.008 ng/ml (HR, 10.055; 95% confidence interval, 5.005–20.200). From the 56 cases that were RM1, 41 cases (73.2%) exhibited a PSA nadir <0.008 ng/ml. There were 42 cases (75.0%) in which only one site was identified to be RM1; among these cases, no significant difference was observed between a PSA level <0.008 ng/ml and a PSA level ≥0.008 ng/ml at the RM1 site (apex, P=0.1460; base, P=0.1384; anterior, P=0.3870; and posterolateral, P=0.5040). There were 14 cases (25.0%) in which multiple sites were RM1; these cases were classified by the number of sites that were RM1 (one vs. multiple) and no significant difference was observed between a PSA level <0.008 ng/ml and a PSA level ≥0.008 ng/ml (P=0.6090). Based on these results, an RM1 of an excised specimen may not reflect the true margin in patients that are treated with RP, specifically in cases where the PSA level is identified to decrease to below the postoperative measurement threshold value (PSA nadir <0.008 ng/ml).

## Introduction

Prostate-specific antigen (PSA) is considered to be an effective prostate tumor marker. The value of PSA as a prostate tumor marker is exhibited particularly well when used to monitor patients following treatment, wherein the majority of follow-up analysis is determined by PSA alone. Specifically, the PSA nadir value of patients that underwent a radical prostatectomy (RP) becomes theoretically zero or demonstrates a very low concentration due to the lack of prostate tissue.

By contrast, the PSA nadir value, which indicates the limit of sensitivity of the measurement kit used, theoretically demonstrates the presence of residual prostate tissue in patients exhibiting benign or malignant disease, including distant metastasis.

The detection limit of recent PSA kits has increased when compared with conventional PSA kits; a third-generation high-sensitivity PSA kit, which was successfully used in a study by Yu *et al* (1) was adopted at the National Kyushu Cancer Center (Fukuoka, Japan) thereby enabling PSA detection at levels <0.008 ng/ml. This enabled determination of the presence of particularly small prostate tissue remains in patients following RP, which usually cannot be detected by computed tomography, magentic resonance imaging or bone scintigraphy from PSA values.

Numerous clinicopathological factors have been proposed to predict biochemical failure (BCF) following RP, including a positive resection margin (RM1), preoperative PSA, clinical stage, Gleason score, extraprostatic extension, seminal vesicle invasion, positive lymph nodes and positive surgical margins among others (2–4). Many of these clinicopathological factors may be associated with biological aggressiveness and potentially with distant metastasis.

Theoretically, a RM1 indicates that there are prostate cancer remains, which affect the PSA value. However, even when the resection margin is positive, there are numerous cases in which the postoperative PSA value declines to less than the measuring limit value and BCF is not detectable. Therefore, to demonstrate that the resection stump of an excised specimen does not necessarily indicate the true resection margin, a retrospective investigation was conducted using the clinicopathological data of patients that underwent an RP, but had not received preoperative treatment.

## Materials and methods

### Tissue specimens

Tissue samples, including embedded whole-mount antegrade RP specimens, obtained from 370 histopathological cases of adenocarcinoma, between September 2003 and March 2011 at the National Kyushu Cancer Center (Fukuoka, Japan) were reviewed. Ninety-five patients were excluded from the investigation, due to a history of receiving hormonal therapy or insufficient preoperative clinical data. The remaining 275 subjects were Japanese and the median postoperative follow-up period was 53.8 months. The median age of the patients was 66 years (range, 47–77 years) and the median preoperative PSA level was 7.874 ng/ml (range, 0.959–39.413 ng/ml). The RP specimens were fixed in 15% neutral-buffered formalin (Wako Pure Chemical Industries, Ltd., Osaka, Japan) for 48–96 h. Whole-organ prostate specimens were serially sectioned perpendicular to the rectal surface at 5-mm intervals. The sections that were predominantly caudal and cephalic were cut in the sagittal plane at 5-mm intervals to assess the bladder neck and apical margins. The specimens were embedded in paraffin, cut into 5-μm sections and stained with hematoxylin and eosin. RP specimens were routinely evaluated with respect to the margin status at the prostatic apex (distal margin), base (proximal margin at the bladder neck) and circumferentially around the organ, with the latter categorized broadly into anterior, posterior, lateral and posterolateral segments. A positive surgical margin was defined as a tumor that extended to the surface of the prostate wherein the surgeon was required to cut across the tissue plane (5,6). A pathologist evaluated the degree of malignancy of the prostatectomy specimens, according to the 2005 grading system proposed by the International Society of Urological Pathology (7), and the pathological stage, based on the 2009 tumor, node, metastasis classification (8). In the present study, the PSA nadir was defined as the lowest PSA value following an RP, with a measurement limit of the serum PSA value <0.008 ng/ml. The definition of BCF following an RP included two consecutive observations of PSA values ≥0.2 ng/ml (9). All patients provided written informed consent. This study was approved by the ethics committee of the National Kyushu Cancer Center (Fukuoka, Japan).

### Statistical analysis

Statistical analyses were conducted using the JMP^®^ Pro version 9.0.2 software package (SAS Institute, Inc., Cary, NC, USA). The significance of the clinicopathological parameters associated with BCF was assessed using the Cox proportional hazards regression model. The Mann-Whitney U test and χ^2^ test were used to determine differences between the patients with a PSA nadir <0.008 ng/ml and those with a PSA nadir of ≥0.008 ng/ml. P<0.05 was considered to indicate a statistically significant difference.

## Results

### Patient clinicopathological profiles

The profiles of the analyzed patients are presented in Table I. The number of clinical stage ≥T2, and pathological stage ≥T3 patients was 151 (54.9%) and 100 (36.4%), respectively. The number of patients with a biopsy Gleason score of ≥8 and an RP Gleason score of ≥8 was 71 (25.8%) and 60 (21.8%), respectively. The number of patients with extraprostatic extension, RM1s, seminal vesicle invasion and positive lymph nodes was 92 (33.5%), 56 (20.4%), 13 (4.7) and 3 (1.1%), respectively. In addition, the serum PSA levels fell to <0.008 ng/ml in 239 patients (86.9%) following surgery.

### Correlations between patient characteristics and the incidence of BCF (Table II)

The correlations between patient characteristics and the incidence of BCF are presented in Table II. According to the Cox proportional hazards analysis, preoperative characteristics, including the preoperative PSA level, clinical tumor stage and biopsy Gleason score, were found to be significant predictors of BCF. Additionally, based on the univariate analysis, the postoperative characteristics, such as the pathological tumor stage (pT), RP Gleason score, extraprostatic extension, RM1s, seminal vesicle invasion, positive lymph nodes and the PSA nadir, were identified to be significant predictors. According to the multivariate analysis, statistically significant differences were identified in the preoperative PSA level, pT, RP Gleason score and a PSA nadir <0.008 ng/ml (P=0.0065, 0.0006, 0.0002 and <0.0001, respectively). The parameter with the highest hazard ratio was a PSA nadir <0.008 ng/ml (HR 10.055; 95% confidence interval, 5.005–20.200).

### RM1 location and quantity stratified by the PSA nadir (<0.008 ng/l versus ≥0.008 ng/ml)

A PSA nadir <0.008 ng/ml was the result observed in 41 (73.2%) out of the 56 RM1 cases (Table III). In the present study there were 42 cases (75.0%) with only one RM1 site, among which the apex was observed to be the most common (n=24) followed by anterior (n=11); lateral and posterior RM1 sites were not observed in any cases. With regard to the cases in which there was only one site that was RM1, no significant difference was observed between a PSA level <0.008 ng/ml and a PSA level ≥0.008 ng/ml (apex, P=0.1460; base, P=0.1384; anterior, P=0.3870; and posterolateral, P=0.5040). There were 14 cases (25.0%) in which multiple sites were RM1, among which apex + anterior was the most common, accounting for eight cases. The cases were classified according to the number of sites that were RM1 (one vs. multiple) and no significant difference was observed between a PSA level <0.008 ng/ml and a PSA level ≥0.008 ng/ml (P=0.6090).

## Discussion

No other tumor marker has been determined to be as efficacious in the diagnosis, management and treatment of a disease as the PSA level in the setting of prostate cancer.

The ectopic expression of PSA has been reported in smaller concentrations in the tissue of malignant breast tumors (10), normal breast tissue (11,12), breast milk (13), female serum (12), and adrenal and renal carcinomas (14); however, for practical and clinical purposes, PSA is organ-specific and is primarily produced by prostatic luminal epithelial cells (13,15,16). Although it is organ-specific, PSA is not cancer-specific, as demonstrated by the substantial overlap in values observed between males with benign versus malignant prostate disease (17,18).

The PSA level is expected to be undetectable within six weeks of a successful RP (20). Thus, a persistently elevated PSA level indicates the presence of PSA-producing tissue within the body. In patients treated with RP, such findings are generally hypothesized to reflect residual cancer due to the presence of either micrometastases (that were not detected or were undetectable prior to surgery) or residual disease in the pelvis, potentially due to an RM1 (9). Yu *et al* (1) classed reagents with an ultrasensitive PSA detection threshold value of 0.1–0.3 ng/ml as first-generation agents, those with a value of 0.02–0.1 ng/ml as second-generation agents and those with a value of 0.001–0.02 ng/ml as third-generation agents. Due to the development of such a highly sensitive PSA reagent, even smaller values became measurable following RP when compared with conventional reagents, thereby enabling clinicians to observe the presence of PSA-producing tissues regardless of whether they are benign or malignant and even when PSA is present in particularly small quantities.

At the National Kyushu Cancer Center, ultrasensitive PSA was used during the follow-up period in patients that had undergone an RP between September 2003 and March 2011, with a measuring limit value of <0.008 ng/ml. Accordingly, when the value drops to less than the postoperative measuring limit, it may be proposed that the PSA-producing tissues do not theoretically exist, or only particularly small PSA-producing tissues (<0.008 ng/ml) are present.

As shown in Table I, the PSA nadir fell to <0.008 ng/ml in 239 cases (86.9%) among the 275 patients undergoing RP in the present study, while BCF was noted in 43 cases (15.6%). In a previous study, Ellis *et al* (21) reported the PSA value to be below the measurement sensitivity level of 0.008 ng/ml in 86.2% of cystoprostatectomy cases in which prostate cancer was not detected, thus indicating that RP was being performed appropriately at the National Kyushu Cancer Center.

Generally, in surgical oncology, a wider excision margin is associated with a lower likelihood of residual disease. This concept may be applied to organs that are present in abundance, such as the bowel. In urology, for example, Khalifeh *et al* (22) reported that the cases exhibiting positive surgical margins, among patients treated via robot-assisted partial nephrectomy, displayed higher rates of recurrence and metastasis (P<0.001).

As indicated in Table IIA, univariate analysis demonstrated RM1 as a significant factor affecting BCF (P=0.0005). However, according to the multivariate analysis (Table IIB), significant factors affecting BCF included the preoperative PSA levels, pT, RP Gleason score, and PSA nadir, with RM1s not identified as a significant factor affecting BCF. A total of 56 of 275 cases (20.4%) were RM1, among which BCF was observed in only 17 cases (30.4%; Table I).

According to a study by Epstein *et al* (23), although surgical margin is a significant factor, the recurrence rate is ~30–50% even when the margin is positive; however, not all cases recur, with no significant difference from the results of the present study. Therefore, a comparative investigation was conducted for the PSA nadir value in each RM1 case. As demonstrated in Table III, the postoperative PSA value decreased to less than the measurement sensitivity (0.008 ng/ml) in 41 cases (73.2%) out of all of the RM1 cases. A comparative investigation was initially performed between the PSA nadir <0.008 ng/ml and the PSA nadir ≥0.008 ng/ml groups from among all 42 cases (75.0%) in which only one site was RM1; however, there was no significant difference observed between RM1 location in the two groups (apex, P=0.1460; base, P=0.1384; anterior, P=0.3870; and posterolateral, P=0.5040). Subsequently, a comparative investigation was conducted between the PSA nadir <0.008 ng/ml and the PSA nadir ≥0.008 ng/ml groups for the quantity of sites that were RM1 (one vs. multiple); however, no significant difference was observed (P=0.6090). Therefore, it is proposed that an RM1 exhibited by an RP specimen may not truly reflect an RM1 within a patient.

During surgery, care must be taken when extracting a tumor, such that it does not become RM1. For example, in the area of urology, with regard to radical nephrectomies for renal cancer, resections are conducted with sufficient attention given to Gerota’s fascia, which is the loose connective tissue that connects to the renal cancer. In addition, in the case of a partial nephrectomy, the resection is conducted concurrently with ascertaining the border of the tumor during the surgical procedure, such that it does not become margin-positive, by leaving a certain degree of surgical margin. This is due to the hypothesis that when a resection margin is histopathologically diagnosed as positive, the remaining cancer tissue in the distal side of the resection margin may lead to recurrence (24). A feature of prostate cancer is the presence of multiple adenocarcinoma foci, which has been observed in 50–76% of all RP specimens in previous studies (25,26). As a result, RP is considered to be the gold standard for surgical treatment. Furthermore, surgery cannot be performed while ascertaining the border between the tumor and the normal tissues (as in renal cancer), therefore, surgery is conducted with reference to preoperative rectal examinations and imaging analysis.

In the cases described in the present study, where the tissue required removal with an enlarged margin (based on the results of a preoperative examination), the intention was to remove the prostate with adequate periprostatic tissue compared with the quantity observed in routine RP. Briefly, dissection of the dorsal vein complex was conducted adequately distally from the anterior surface of the prostate, while applying downward pressure on the prostate; in addition, wide resection of the bladder neck was performed more cranially. In the posterior region, Denonvilliers’ fascia was dissected more dorsally and in the posterolateral region, dissection of the prostatic pedicle was conducted more dorsally after separating the rectum more dorsally from the prostatic pedicle, without sparing the neurovascular bundles.

However, two elements are required to perform a successful RP against prostate cancer; the first element concerns the radicality of the cancer. As much of the surrounding tissues as possible should remain attached to the prostate to further reduce the ratio of the RM1 and thus reduce the potential for recurrence. However, the other element, which conflicts with this concept, is that function preservation (such as urinating and sexual function) is required. Therefore, the extraction range cannot be expanded and tissues may only be extracted within the range in which the urine function, in particular, may be preserved.

Theoretically, with regard to localized prostate cancer, it is hypothesized that there are two associations between the RM1 and the postoperative PSA nadir value of the excised specimen. The first is that the resection margin becomes pathologically positive due to the prostate cancer invading outside the surgical resection line, which prevents the PSA nadir value from declining to less than the measurement sensitivity. The second is that the prostate cancer does not invade outside of the surgical resection line, however, the resection margin becomes pathologically positive as a result of the surgical procedure, which leads to the PSA nadir value decreasing to less than the measurement sensitivity.

*In vivo*, the prostate should be surrounded by neighboring connective tissues and organs, with the prostate tissues exposed at the resection margin of the excised specimen due to its removal during the the surgical procedure. Furthermore, the useful tumor marker, PSA, may be used to understand the presence of particularly small prostate tissue remains following RP, whether they are cancerous or not.

In conclusion, regarding cases in which the postoperative levels of ultrasensitive PSA decrease to less than the measurement sensitivity (i.e. <0.008 ng/ml), although the excision specimen appears to be RM1, this may not necessarily truly indicate that the resection margin within the patient is positive.

## Figures and Tables

**Table I:** Patient clinicopathological profiles.

Characteristic	Total	BCF (−)	BCF (+)
Patients, n (%)	275 (100)	232 (84.4)	43 (15.6)
Median age, years (range)	66 (47–77)	66 (47–77)	69 (51–75)
Median preoperative PSA, ng/ml (range)	7.874 (0.959–39.413)	7.437 (0.959–39.413)	10.347 (5.024–39.123)
Clinical stage, n (%)			
cT1c	124 (45.1)	114 (49.1)	10 (23.3)
≥cT2	151 (54.9)	118 (50.9)	33 (76.7)
Biopsy Gleason score, n (%)			
≤7	204 (74.2)	182 (78.4)	22 (51.2)
≥8	71 (25.8)	50 (21.6)	21 (48.8)
Pathological stage, n (%)			
≤pT2	175 (63.6)	163 (70.3)	12 (27.9)
≥pT3	100 (36.4)	69 (29.7)	31 (72.1)
Radical prostatectomy Gleason score, n (%)			
≤7	215 (78.2)	191 (82.3)	24 (55.8)
≥8	60 (21.8)	41 (17.7)	19 (44.2)
Extraprostatic extension, n (%)			
0	183 (66.5)	168 (72.4)	15 (34.9)
1	92 (33.5)	64 (27.6)	28 (65.1)
Resection margin, n (%)			
0	219 (79.6)	193 (83.2)	26 (60.5)
1	56 (20.4)	39 (16.8)	17 (39.5)
Seminal vesicle invasion, n (%)			
0	262 (95.3)	225 (97.0)	37 (86.0)
1	13 (4.7)	7 (3.0)	6 (14.0)
Positive lymph nodes, n (%)			
0	272 (98.9)	231 (99.5)	41 (95.3)
1	3 (1.1)	1 (0.5)	2 (4.7)
PSA nadir, n (%)			
<0.008 ng/ml	239 ( 86.9)	216 (93.1)	23 (53.5)
≥0.008 ng/ml	36 (13.1)	16 (6.9)	20 (46.5)

Clinical and pathological staging was based on TNM staging (2009) (8). BCF, biochemical failure (two consecutive PSA values ≥0.2 ng/ml); PSA, prostate-specific antigen; (−), absence of BCF; (+), presence of BCF; 0, negative; 1, positive.

**Table II:** Correlations between patient characteristics and incidence of biochemical failure.

A, Univariate analysis			

Characteristic	Hazard ratio	P-value	95% CI
Age	1.009	0.7353	0.961–1.062
Preoperative PSA	1.079	<0.0001	1.042–1.112
cT1 vs. ≥cT2	3.121	0.0006	1.593–6.690
Biopsy Gleason score, ≤7 vs. ≥8	3.327	0.0002	1.811–6.099
pT2 vs. pT3	5.413	<0.0001	2.852–10.976
RP Gleason score, ≤7 vs. ≥8	3.155	0.0004	1.703–5.763
EPE 0 vs. EPE 1	4.226	<0.0001	2.290–8.120
RM 0 vs. RM 1	3.239	0.0005	1.714–5.975
Sv 0 vs. Sv 1	6.227	0.0009	2.332–13.978
pN0 vs. pN1	6.701	0.0423	1.087–22.005
PSA nadir, <0.008 vs. ≥0.008 ng/ml	11.606	<0.0001	6.193–21.645

B, Multivariate analysis			

Characteristic	Hazard ratio	P-value	95% CI

Preoperative PSA	1.064	0.0065	1.018–1.113
pT2 vs. pT3	3.312	0.0006	1.666–6.583
RP Gleason score, ≤7 vs. ≥8	3.339	0.0002	1.772–6.293
PSA nadir, <0.008 vs. ≥0.008 ng/ml	10.055	<0.0001	5.005–20.200

Clinical and pathological staging was based on the TNM classification (2009). PSA, prostate-specific antigen; cT, clinical tumor stage; pT, pathological tumor stage; RP, radical prostatectomy; EPE, extraprostatic extension; RM, resection margin; Sv, seminal vesicle invasion; pN, pathological lymph node stage ; CI, confidence interval; 0. negative; 1, positive. P<0.05 was considered to indicate a statistically significant difference.

**Table III:** RM1 location and quantity stratified by the level of PSA nadir (<0.008 vs. ≥0.008 ng/ml).

		PSA nadir, ng/ml		
			
Variable	Total	<0.008	≥0.008	P-value
Patients, n	56	41	15	
RM1 location				
Apex	24	15	9	0.1460
Apex + anterior	8	7	1	
Apex + posterolateral	2	0	2	
Apex + base	2	2	0	
Base	5	5	0	0.1384
Base + anterior	1	1	0	
Base + posterolateral	1	1	0	
Anterior	11	9	2	0.3870
Posterolateral	2	1	1	0.5040
Lateral	0			
Posterior	0			
RM1 quantity				
One	42	30	12	0.6090
Multiple	14	11	3	

RM1, positive resection margin; PSA, prostate-specific antigen.
